# Exercise for Non‐Motor Symptoms of Parkinson’s Disease: An Evidence‐Based Review of Clinical Trials, Dosing Considerations, and Mechanistic Insights

**DOI:** 10.1155/np/7191029

**Published:** 2026-04-01

**Authors:** Shuang Hu, Jing Lei, Hao-Jun You

**Affiliations:** ^1^ Center for Translational Medicine Research on Sensory-Motor Diseases, Yan’an University, Yan’an, 716000, China, yau.edu.cn; ^2^ Key Laboratory of Pain Precision Treatment and Rehabilitation of Shaanxi Province Higher Education Institutions, Yan’an, 716000, China

**Keywords:** exercise intervention, neuroimmune modulation, neuroplasticity, non-motor symptoms, Parkinson’s disease

## Abstract

Parkinson’s disease (PD) is a neurodegenerative disorder characterised primarily by motor symptoms. However, non‐motor symptoms (NMS) also substantially affect patients’ quality of life. In recent years, exercise therapy has attracted increasing attention as a complementary treatment for PD and is regarded as a promising strategy for alleviating neuropsychiatric symptoms. A substantial body of evidence suggests that exercise improves multiple NMS domains in PD, and this effect may be achieved through several mechanisms. Despite the growing recognition of its benefits, the evidence base is limited by small sample sizes, heterogeneous interventions and inconsistent outcome measures. This underscores the necessity for further research to be conducted into the distinct effects of various forms of exercise. The present review synthesises the clinical evidence relating the impact of different exercise modalities on mood symptoms (anxiety and depression), cognitive dysfunction, sleep disturbances, pain and autonomic dysfunction in PD. It also discusses how exercise parameters such as intensity and duration can regulate NMS, exploring their potential mechanisms and clinical applications. These insights may inform new approaches to the comprehensive management of PD and future therapeutic strategies targeting NMS.

## 1. Introduction

Parkinson’s disease (PD) is a prevalent neurodegenerative disorder, characterised by the progressive loss of dopaminergic neurones and the presence of hallmark motor symptoms, including resting tremor, bradykinesia, rigidity and postural instability [1]. It is evident that, in addition to motor manifestations, a significant proportion of patients (over 90%) experience non‐motor symptoms (NMS) that result in considerable impairment to quality of life and daily functioning. These symptoms encompass a wide range of conditions, including emotional disturbances (e.g. anxiety and depression), sleep disorders, cognitive impairment, pain and autonomic dysfunction. It is noteworthy that many of these symptoms can occur during the prodromal phase and have the capacity to affect psychological and physiological well‐being [2]. Consequently, early recognition and effective management of NMS are critical for improving patient outcomes.

Pharmacological therapy remains central to the management of PD, but it is often limited by adverse effects and suboptimal efficacy for NMS [2]. It is evident that exercise has gained prominence as a significant adjunct strategy, with mounting evidence underscoring its benefits across multiple NMS domains, including mood, sleep, cognition, and pain [3]. Contrary to previous reviews that offer broad narrative summaries or merely catalogue mechanistic findings, this review synthesises the highest‐level clinical evidence within each major NMS domain (accompanied by a cross‐domain summary), with the aim of enhancing clinical interpretability while minimising redundancy. Given the paucity of mechanistic evidence in this field, we propose a hypothesis‐driven peripheral–central framework to account for the convergent exercise‐related benefits observed across NMS domains. This framework also highlights key translational gaps (e.g. standardised NMS phenotyping and dose–response parameters) that constrain the precision of exercise prescriptions. The present study, therefore, examines the effects of exercise interventions on NMS in PD and discusses potential underlying mechanisms to inform clinical application and comprehensive disease management.

## 2. Methods

This narrative review synthesised current evidence on the effects of exercise on NMS in PD. It also summarised preclinical findings solely in terms of biological context. In view of the considerable heterogeneity observed in study designs, exercise modalities, and outcome measures, it was determined that a narrative approach would be a more suitable form of analysis than a systematic review [4]. A structured PubMed search was conducted from December 2015 to January 2026, with a focus on studies published in the most recent 5 years. Additionally, the reference lists of key reviews and clinical trials were screened. A comprehensive search strategy was employed, encompassing search terms such as ’Parkinson’s disease’, ’exercise/physical activity’, ’aerobic’, ’resistance training’, ’mind–body’, ’non‐motor symptoms’, ’depression/anxiety’, ’cognition’, ’sleep’, ’pain’, and ’autonomic dysfunction’.

Eligible studies were human clinical investigations (meta‐analyses, randomised/controlled trials and cohort studies) reporting at least one NMS outcome following a defined exercise intervention; pilot or pre–post studies were included when higher‐level evidence was limited for a domain. A comprehensive review of the extant literature was conducted to inform mechanistic hypotheses, with the caveat that the findings could not be used to support conclusions on clinical efficacy. The evidence was then organised according to level of evidence, with meta‐analyses and randomised controlled trials given the highest priority, followed by controlled and observational studies and finally pilot studies. This evidence was synthesised by NMS domain and exercise modality/dose when reported.

Given the narrative design and variability in interventions and endpoints, there is potential for selection and publication bias; many of the included trials are small and differ in blinding, adherence monitoring and outcome definitions, which limits cross‐study comparability.

## 3. NMS in PD: Definition and Temporal Course

### 3.1. Definition and Clinical Spectrum

The neuropsychiatric, cognitive, sleep/circadian, autonomic and sensory manifestations encompassed by NMS in PD are not captured by classical motor diagnostic criteria. From a clinical perspective, NMS are frequently categorised into the following five distinct groups: (i) neuropsychiatric symptoms (e.g. depression, anxiety, apathy and hallucinations/psychosis); (ii) cognitive impairment; (iii) sleep and circadian disturbances (e.g. insomnia, excessive daytime sleepiness and REM sleep behaviour disorder); (iv) autonomic dysfunction (gastrointestinal, urinary, cardiovascular, sexual and thermoregulatory symptoms) and (v) sensory symptoms including pain and fatigue [5, 6]. In order to facilitate systematic recognition and reporting in research and practice, the development of structured screening tools such as the Non‐Motor Symptoms Questionnaire (NMS Quest) has been undertaken, with the objective of highlighting the breadth of NMS burden across disease stages [7].

### 3.2. Temporal Course of NMS

The progression of PD follows a staged trajectory (preclinical → prodromal → clinical PD), and several NMS‐related features are explicitly incorporated into the Movement Disorder Society (MDS) research criteria for prodromal PD [8, 9]. The early non‐motor manifestations that are most frequently reported include REM sleep behaviour disorder, hyposmia, constipation, mood disturbances and autonomic complaints. By contrast, cognitive impairment, complex pain phenotypes and more severe autonomic dysfunction often become increasingly prominent with disease progression and multisystem involvement [6, 9]. This temporal heterogeneity suggests that the timing, modality and dose of exercise interventions may need to be aligned with specific symptom domains and disease stage. Accordingly, the present review is structured by major NMS domains and prioritises clinical evidence, while mechanistic findings are synthesised as hypothesis‐generating support and are explicitly separated from clinical efficacy claims.

## 4. Impact of Exercise on Different Types of NMS

According to the World Health Organization (WHO), ’exercise’ is defined as a subcategory of physical activity that is planned, structured, repetitive and purposeful, with the objective of improving or maintaining one or more components of physical fitness. In this review, the term ’exercise’ is understood to encompass prescribed and supervised forms of aerobic exercise (e.g. walking, treadmill training and cycling); resistance and strength training and structured mind–body programmes (e.g. Tai Chi, Qigong and yoga/meditation‐based interventions), provided that the exercise dose (frequency, intensity, duration and type) is specified.

As demonstrated in Table 1, there is a paucity of clinical evidence to support the hypothesis that physical exercise exerts an effect on NMS in cases of PD.

**Table 1:** Clinical evidence of the effects of exercise on non‐motor symptoms (NMS) in Parkinson’s disease.

NMS domain	Evidence level	Study design	Exercise modality	Dose and intensity (FITT/definition)	Population/demographics	Sample size (*n*)	Primary clinical outcome (*s*)	Main finding (direction)	Ref.
Mood (depression/anxiety)	Level I	Systematic review and meta‐analysis	Exercise (various)	NA (varies across included trials)	PD (varies across included trials)	11 studies (depression), *n* = 728; 6 studies (anxiety), *n* = 241	Depression/anxiety symptom scales (varies)	Pooled benefit reported	[10]
Mood (depression/anxiety)	Level I	Meta‐analysis	Exercise (various)	NA (varies across included trials)	PD (varies across included trials)	19 RCTs; total *n* = 1302	Depressive symptoms (varies)	Pooled benefit reported	[11]
Mood (depression/anxiety)	Level II	Randomised clinical trial	Mindfulness yoga vs. stretching/resistance	8 weeks; weekly MY 90 min; weekly SRTE 60 min; +home practice 20 min twice/week	PD (H&Y 1–3); mean age 63.8 ± 7.1 years; 56% female	138 (MY 71; SRTE 67)	Anxiety/depression; HRQOL	Anxiety/depressio ↓ vs. comparator	[12]
Cognition	Level I	Systematic review and meta‐analysis of RCTs	Exercise (various)	NA (varies across included trials)	PD (age range across studies: 50.3–75.5 years)	21 RCTs; total *n* = 761	Global cognition/executive domains (varies)	Modest group‐level benefit; heterogeneity noted	[13]
Cognition	Level II	Randomised controlled trial	Tai Chi vs. aerobic exercise	12 weeks; AE 3 ×/week, 30 min/session (recumbent bike; 8× [1 min at 70%–75% HRR + 2 min active recovery], RPE 9–11); TC 2 ×/week, 60 min/session	Early‐stage PD (modified H&Y 1–2; age 50–80 years; on‐medication during assessments)	56 randomised (AE 18; TC 20; control 18); analysed *n* = 43 (AE 14; TC 16; control 13)	Motor + neurocognitive performance	Modality‐specific patterns of benefit reported	[14]
Cognition	Level II	Controlled/long‐term intervention study	Tai Chi vs. aerobic exercise	12 weeks; AE 3 ×/week, 30 min/session (recumbent bike; 70%–75% HRR); TC 2 ×/week, 60 min/session	Early‐stage PD (modified H&Y 1–2; medically stable)	61 (AE 20; TC 21; control 20)	Antioxidant activity; cognitive function	Improvement reported; between‐modality differences described	[15]
Sleep disorders	Level I	Systematic review and meta‐analysis	Exercise (various)	NA (varies across included trials)	Adults with PD (>18 years; varies)	12 controlled trials (10 RCT + 2 non‐randomised); total *n* = 690	Sleep quality (e.g. PDSS/PSQI; varies)	Pooled improvement in subjective sleep quality reported	[16]
Sleep disorders	Level II	Randomised controlled trial	Exercise intervention	16 weeks; supervised exercise training 3 ×/week (combined resistance + body‐weight functional mobility; sessions before 2 PM)	PD (H&Y 2–3; age > = 45 years; not in regular exercise programme)	71 randomised; completed/protocol *n* = 55 (EX 27; SH‐C 28)	Objective + subjective sleep (including PSG)	Improvement reported on sleep outcomes (domain‐specific)	[17]
Sleep disorders	Level II	Clinical trial (resistance training)	Progressive resistance training	12 weeks; progressive resistance training 2 ×/week (linear periodisation)	Moderate PD (H&Y 2–3; age 50–80 years; stable dopaminergic therapy)	PD *n* = 22 (RT 11; control 11); + healthy controls *n* = 31 (baseline only)	Sleep quality measures	Improved sleep quality reported	[18]
Pain	Level II–III	Randomised pilot trial (subgroup analysis)	Cycling exercise	24 weeks; supervised cycling 3 ×/week, 40–60 min/session (duration + 10 min every 8 weeks)	Early PD (age 50–80 years; H&Y 1–2; disease duration <5 years)	33 randomised (cycling 22; control 11); subgroup analysis *n* = 25 (cycling 16; control 9)	Pain types/PD‐specific pain measures	Subtype‐specific improvement reported	[19]
Pain	Level II	Randomised clinical trial	Aquatic therapy	10 weeks; Ai Chi aquatic programme 2 ×/week (per protocol)	PD (H&Y 1–3; age >40 years; off‐medication; MMSE ≥ 24; stable meds ≥ 4 weeks)	30 (Ai Chi 15; control 15)	Pain intensity + function	Pain ↓; function reported ↑	[20]
Pain	Level III	Cohort/controlled exercise comparison	Flexibility/relaxation; walking; Nordic walking	6 months; 3 ×/week, 70 min/session (NW vs. walking vs. flexibility/relaxation)	PD (H&Y II–III; UK Brain Bank criteria)	90 randomised (3 groups)	Pain‐related symptoms	Improvement reported across programmes	[21]
Autonomic dysfunction	Level II	Randomised controlled trial	Progressive resistance training	12 weeks; RT 2 ×/week; 5 exercises; 2–4 sets of 12–6 RM (5‐min treadmill warm‐up)	PD (age ≥ 50 years; modified H&Y 2–3; assessed in ’on’ medication state)	30 PD (PDT vs. PDC); + matched healthy controls	Cardiovascular autonomic regulation (HRV); orthostatic response	Signal of benefit reported on autonomic measures	[22]
Autonomic dysfunction	Level III	Pilot study	Strength training (leg‐focused)	8 weeks; 2 ×/week; 45–60 min/session (20‐min warm‐up + 20‐min leg strength + 20‐min balance; +home calf exercises 3 ×/week)	PD with orthostatic hypotension (MoCA ≥ 24; sufficient mobility)	29 randomised (cross‐over groups A 15; B 14)	Orthostatic blood pressure outcomes	Feasibility/effects assessed; limited evidence	[23]
Autonomic dysfunction	Level III	Prospective rehab/secondary analysis	Supervised aerobic rehabilitation	3 months; start 20 min 3 ×/week, progress by week 4 to 150 min/week (30 min 5 ×/week or 50 min 3 ×/week); intensity 50%–60% HRmax to 70%–80%	PD (age 45–75 years; H&Y 2–3; stable PD meds ≥ 3 months)	110 (55 male; 55 female)	Heart rate variability (HRV)	Exercise‐associated HRV changes reported	[24]
Cross‐domain (NMS)	Level III	Clinical review	Exercise (overview)	NA	PD	NA	Multiple NMS domains	Summarises evidence across NMS domains	[25]

Abbreviations: AE, aerobic exercise; ANS, autonomic nervous system; BDI‐II, Beck Depression Inventory‐II; BMI, body mass index; CG, control group; CONSORT, Consolidated Standards of Reporting Trials; ECG, electrocardiogram; EX, exercise group; FITT, frequency–intensity–time–type; H&Y, Hoehn and Yahr stage; HADS, Hospital Anxiety and Depression Scale; HC, healthy control; HF, high frequency; HIIT, high‐intensity interval training; HR, heart rate; HRmax, maximal heart rate; HRQOL, Health‐Related Quality of Life; HRR, heart rate reserve; HRV, heart rate variability; Level I, meta‐analysis/systematic review; Level II, randomised/controlled trials; Level III, cohort/pilot/pre–post/secondary analyses; LF, low frequency; MICT, moderate‐intensity continuous training; MMSE, Mini‐Mental State Examination; MoCA, Montreal Cognitive Assessment; MY, Mindfulness yoga; NA, not applicable; NMS, non‐motor symptoms; NR, not reported; NW, Nordic walking; OH, orthostatic hypotension; PD, Parkinson’s disease; PDC, PD control group; PDSS, Parkinson Disease Sleep Scale; PDT, PD training group; PSG, polysomnography; PSQI, Pittsburgh Sleep Quality Index; RCT, randomised controlled trial; REM, rapid eye movement; RM, repetition maximum; RM‐ANOVA, repeated‐measures ANOVA; RPE, rating of perceived exertion; RT, resistance training; SH‐C, sleep hygiene control; SRTE, stretching and resistance training exercises; TC, Tai Chi.

### 4.1. Clinical Evidence

#### 4.1.1. Mood Disorders (Depression and Anxiety)

The efficacy of exercise as a therapeutic intervention for the alleviation of anxiety and depressive symptoms in PD is supported by clinical studies. Exercise modalities encompass aerobic exercise, resistance training and mind–body practices such as yoga, meditation, Tai Chi and Qigong. A number of studies have reported that yoga reduces anxiety and depressive symptoms in individuals with PD and improves health‐related quality of life (HRQOL) [26–28]. A growing body of research has demonstrated that mind–body exercise interventions, such as meditation and yoga, have been associated with improvements in anxiety and depression, as well as with reductions in stress‐ and inflammation‐related biomarkers (e.g. IL‐6, cortisol and TNF‐α) [26].

In a randomised controlled study, Kwok et al. assigned 138 patients with PD to either an 8‐week mindfulness yoga programme or a conventional stretching exercise programme. The results demonstrated that mindfulness yoga was more effective than traditional stretching exercise in alleviating anxiety and depressive symptoms [28]. Evidence has demonstrated that both mind–body exercises and resistance training have the capacity to significantly improve emotional symptoms in patients diagnosed with PD [10]. A number of studies have indicated that mindfulness yoga is more efficacious in reducing anxiety and depression than conventional resistance training [12]. Conversely, the impact of stand‐alone aerobic exercise on depressive symptoms appears to be negligible. Conversely, the efficacy of combined exercise programmes has been demonstrated to be more substantial [11]. Taken together, these findings suggest that the type of intervention (particularly the mind–body or multimodal components) may be an important determinant of mood‐related outcomes in PD.

#### 4.1.2. Cognitive Dysfunction

A mounting body of clinical evidence has come to support the notion that physical exercise can serve as a non‐pharmacological strategy, one which has been shown to be associated with cognitive benefits in individuals diagnosed with PD. This finding is of particular relevance to the management of neurodegenerative diseases [29]. The accumulation of clinical data, encompassing controlled trials and meta‐analyses, has indicated that exercise can enhance global cognition and specific domains, notably executive function, in individuals diagnosed with PD. Nevertheless, substantial heterogeneity persists across interventions and outcome scales [13].

The findings of clinical studies and syntheses of evidence support the hypothesis that there is a modest improvement in cognitive function in PD with exercise when considered at the group level [13]. The findings of research in this field indicate that long‐term aerobic exercise and Tai Chi have both been reported to reduce oxidative damage and alleviate cognitive impairment in PD, with aerobic exercise appearing more effective than Tai Chi in this respect [15]. A recent longitudinal study has reported the occurrence of exercise‐related improvements in neurocognitive performance in subjects performing Tai Chi Chuan or aerobic exercise in the early stages of PD. These improvements were found to be domain‐ and task‐specific [14]. In addition to aerobic exercise and Tai Chi, resistance training—whether implemented in isolation or in combination with aerobic exercise—has been demonstrated to enhance cognitive function in patients diagnosed with PD [30]. The integration of exercise interventions with other methodologies, including cognitive training, pharmacotherapy, non‐invasive brain stimulation and artificial intelligence technologies, has been documented to result in enhanced improvements in cognitive performance [31]. Overall, these findings suggest that exercise may be a clinically relevant adjunct strategy within comprehensive PD management and underline the need for standardised cognitive endpoints and consistent dose reporting in future trials.

#### 4.1.3. Sleep Disorders

Sleep disorders are prevalent in PD, with their incidence increasing as the disease progresses. As has been documented, a number of sleep‐related issues have been reported, including, but not limited to, insomnia, excessive daytime sleepiness, circadian rhythm disruptions, obstructive sleep apnoea, restless legs syndrome and rapid eye movement sleep (REM) behaviour disorder. The extant research, incorporating systematic reviews and meta‐analyses, suggests that exercise interventions can enhance subjective sleep quality in PD [16, 17, 32, 33].

The findings of clinical research indicate that a variety of exercise modalities may have a beneficial effect on sleep disturbances in individuals diagnosed with PD. Evidence suggests that exercise interventions can help mitigate sleep‐related problems in PD, with moderate‐ to high‐intensity exercise showing greater benefits than low‐intensity exercise [16]. For example, a 12‐week progressive resistance training programme has been shown to substantially increase muscle strength in patients with moderate‐stage PD while also improving sleep quality [18]. A substantial body of research has demonstrated the efficacy of traditional Chinese exercises in enhancing sleep quality, reducing anxiety and depression and promoting cognitive function in individuals diagnosed with PD [34]. It has been documented that Qigong practice exerts a beneficial effect on patients suffering from PD, with reports indicating a reduction in serum TNF‐α levels and an enhancement in the quality of night‐time sleep [35, 36]. Moreover, a 12‐week Tai Chi programme in conjunction with conventional exercise training has been demonstrated to improve both sleep quality and cognitive function in patients with PD [37]. The current body of evidence suggests that exercise therapy may improve sleep disorders in PD; however, further large‐scale, high‐quality clinical trials are still needed to evaluate long‐term efficacy and to determine optimal intervention protocols.

#### 4.1.4. Pain

Pathological pain in PD can be divided into five categories: musculoskeletal pain, dystonic pain, radicular neuralgia, central pain and akathisia [38]. The King’s Parkinson’s Disease Pain Scale (KPPS) is a tool that has been developed and validated for the specific purpose of evaluating and assessing pain in individuals diagnosed with PD. The scale in question has been developed for the purpose of quantifying pain severity and frequency across seven distinct domains. These domains, which are listed below, include the following: the musculoskeletal domain, the chronic domain, the fluctuation‐related domain, the nocturnal domain, the orofacial domain, the discolouration domain, the oedema and swelling domain and the radicular domain [39]. As a non‐pharmacological intervention, exercise is frequently used to alleviate pain in PD.

The findings of clinical studies indicate that physical exertion has the potential to alleviate the pain experienced by individuals suffering from PD and may even lead to improvements in specific types of pain. Recent trials have begun to employ PD‐specific tools, such as KPPS, to quantify the exercise‐related analgesic effects. It has been reported that cycling exercise improves certain pain subtypes in early PD [19]. A number of studies have been conducted in order to examine the impact of Pilates and yoga on low back pain in patients with PD. The findings of these studies indicate that both interventions are capable of effectively alleviating symptoms of low back pain [40, 41]. In a similar vein, aquatic exercise programmes (including aquatic Ai Chi/Tai Chi‐based protocols) have demonstrated efficacy in reducing pain intensity in mild‐to‐moderate PD, as evidenced by findings from randomised clinical trials [20]. A subsequent study of 90 patients with PD reported that flexibility and relaxation training, in conjunction with regular walking and Nordic walking, were each associated with improvements in pain‐related symptoms [21]. In light of the heterogeneity of PD pain phenotypes, a single modality may not be suitable for all patients; multimodal and individualised prescriptions (e.g. combining strengthening, flexibility and aerobic components) may be more appropriate.

#### 4.1.5. Autonomic Dysfunction

Autonomic dysfunction is a prevalent feature of PD, manifesting in a range of gastrointestinal, urinary, sexual, thermoregulatory and cardiovascular symptoms. Among these, constipation, orthostatic hypotension and urinary incontinence are particularly notable. In the clinical context, the efficacy of exercise‐based interventions as adjunctive approaches for autonomic‐related outcomes has been a subject of exploration. However, the extant evidence base remains limited, and the outcome measures employed are heterogeneous. Evidence from a number of studies suggests that cardiovascular autonomic function can be enhanced by resistance training and structured aerobic rehabilitation. For instance, a 12‐week progressive resistance training programme has been documented to enhance cardiac autonomic regulation and the systolic blood pressure response to orthostatic stress in individuals diagnosed with PD [22]. An additional pilot study in patients with PD and orthostatic hypotension evaluated leg muscle strength training and assessed orthostatic blood pressure changes under standardised testing conditions [23]. The utilisation of aerobic exercise programmes for the quantification of autonomic adaptation through heart rate variability (HRV) has been a subject of recent research. A prospective rehabilitation study has reported on HRV changes following a supervised aerobic exercise intervention in PD [24]. A randomised controlled pilot study of Qigong (meditative movement) was conducted to investigate the efficacy of this practice in addressing gastrointestinal symptoms. The study reported persistent improvement in constipation as a secondary outcome. However, other autonomic domains, such as urinary and sexual symptoms, remained unaltered. These findings emphasise the domain‐specific and still inconclusive nature of the current evidence base [25].

A comprehensive review of the extant literature indicates that the available clinical data support the hypothesis that exercise may improve selected autonomic‐related outcomes, particularly in the domains of cardiovascular autonomic regulation and constipation in specific cohorts. However, the findings require further validation through larger trials that employ standardised autonomic endpoints (e.g. Scales for Outcomes in Parkinson’s Disease‐Autonomic, orthostatic blood pressure protocols and HRV metrics) to ascertain the optimal exercise prescriptions and the longevity of the observed effects.

### 4.2. Preclinical Evidence

Across models, exercise appears to engage a peripheral‐to‐central signalling cascade: the first category comprises peripheral ’exerkines’ (e.g. FNDC5/irisin) and systemic immunometabolic shifts. The second category consists of downstream modulation of central neuroimmune and plasticity‐related processes. In an MPTP mouse paradigm, the positive effects of aerobic exercise on cognition have been reported, alongside reduced microglial inflammasome formation via an irisin/NLRP3‐related pathway. Mechanistic perturbations (e.g. blocking irisin signalling) have been shown to attenuate these effects, thus supporting a testable peripheral–microglia–neurogenesis/plasticity route [42].

For mood‐ and stress‐related phenotypes, preclinical literature supports the hypothesis that monoaminergic regulation and neuroimmune signalling act as convergent nodes. The scientific literature suggests that the BDNF‐TrkB signalling within REM‐regulating circuits is implicated in the maintenance of homeostasis during REM sleep. However, the evidence for a direct correlation between this signalling and the improvement in sleep quality in individuals with PD remains inconclusive at present [43]. The experimental literature on exercise‐induced hypoalgesia in both animals and humans supports the involvement of endocannabinoid and opioid‐related analgesic systems (e.g. periaqueductal grey matter‐related mechanisms), providing a biologically coherent axis that could be mapped to PD pain phenotypes [44]. Finally, the evidence suggests a plausible relationship between autonomic dysfunction and exercise‐driven shifts in autonomic balance (HRV/baroreflex‐related control). It is important to note that human PD trials have also demonstrated that resistance training has the capacity to enhance cardiac autonomic regulation. This finding serves to corroborate the preclinical plausibility with clinically measurable endpoints [22].

In conclusion, it is suggested that these findings should be interpreted as hypothesis‐generating rather than as causal evidence for clinical efficacy. This is due to the fact that current PD models incompletely capture the heterogeneity and longitudinal evolution of human NMS [25]. As demonstrated in Table 2, there is preclinical and mechanistic evidence for the effects of exercise on NMS in PD.

**Table 2:** Preclinical and mechanistic evidence of the effects of exercise on non‐motor symptoms (NMS) in Parkinson’s disease.

Mechanistic axis	Evidence type	Model/sample	Exercise exposure	Key mechanistic readout	Linked NMS domain (s)	Ref.
Peripheral → central (irisin/NLRP3)	Preclinical (PD model)	MPTP mouse	Aerobic exercise (NR)	Microglia inflammasome (NLRP3); hippocampal neurogenesis; cognition	Cognition	[42]
REM regulation (BDNF in brainstem)	Preclinical neurophysiology	Rat	Not an exercise trial	Localised BDNF expression in brainstem; REM homeostasis	Sleep	[43]
Endocannabinoid system and exercise	Mechanistic review	Mixed (animal/human)	NA	Endocannabinoid pathways implicated in exercise‐induced hypoalgesia	Pain	[44]
Cross‐species dose translation	Translational (human + PD model)	Early PD humans + Pink1 KO rats	Aerobic parameters matched	HR‐based equivalence of aerobic parameters across species	Dose framework (cross‐domain)	[45]
Muscle–brain axis/myokines	Mechanistic review	Mixed	Resistance training	Myokines; neuroprotective signalling	Cognition/mood (context)	[46]
Irisin neuroprotection	Mechanistic review	Mixed	Exercise‐derived irisin	Multiple roles in neuroprotection	Cross‐domain (context)	[47]
Exercise‐induced myokines in ND	Mechanistic review	Mixed	Exercise	Myokines in neurodegenerative diseases	Cross‐domain (context)	[48]

Abbreviations: BDNF, brain‐derived neurotrophic factor; HR, heart rate; KO, knockout; MPTP, 1‐methyl‐4‐phenyl‐1,2,3,6‐tetrahydropyridine; ND, neurodegenerative diseases; NLRP3: NLR family pyrin domain containing 3; PD, Parkinson’s disease; Pink1 = PTEN, induced putative kinase 1, REM, rapid eye movement.

## 5. Differences in the Effects of Various Types of Exercise

### 5.1. Aerobic Exercise

Aerobic exercise is among the most commonly studied forms of exercise in PD and is typically delivered as walking, low‐ to high‐intensity continuous training, interval‐based aerobic training, cycling or swimming [29, 49]. In addition to the domain‐specific outcomes summarised in Section 4, the topic of aerobic exercise is frequently discussed in terms of its feasibility and translational potential. A cross‐species study that employed heart rate (HR) as a shared parameter between PD rodent models and human patients supported the feasibility of prescribing moderate‐intensity aerobic exercise in early‐stage PD [45]. Combined interventions, such as Tai Chi integrated with aerobic exercise, have been shown to improve cognitive and memory function in patients with PD and enhance their antioxidant capacity [14, 15]. Compared to goal‐based exercise training, aerobic exercise alone has a more pronounced effect on alleviating cognitive impairment in patients with PD [50].

Within the context of aerobic programmes, both combination strategy and intensity appear to modify outcomes. The available evidence suggests that combining aerobic exercise with other modalities may offer broader benefits than aerobic exercise alone [51]. Furthermore, the intensity of exercise has been demonstrated to influence efficacy, with higher‐intensity aerobic interventions frequently reporting more substantial effects. However, it should be noted that findings are not uniform across studies [52]. In practice, the prescription of aerobic exercise should be individualised by mode, intensity and feasibility and may be tailored as part of multimodal programmes to match patient goals and tolerance.

### 5.2. Resistance Exercise

Resistance exercise is defined as physical activity in which muscles generate force against external resistance, with the objective of enhancing muscle mass, strength and endurance [29]. In PD, resistance training is increasingly incorporated into rehabilitation programmes, although most trials have focused on motor outcomes and evidence for non‐motor domains remains comparatively limited [30].

The preponderance of extant evidence is of a clinical nature, with a paucity of preclinical studies. In clinical studies, resistance programmes have been associated with improvements in selected non‐motor outcomes (including anxiety, depressive symptoms and sleep quality), although effects are not consistently superior to other exercise modalities across trials [53–55]. Multicomponent rehabilitation programmes that incorporate resistance training (e.g. higher‐intensity programmes combined with interval‐based elements) may offer broader benefits in specific cohorts; however, comparative evidence remains limited [54, 55].

The mechanisms through which resistance training relates to cognitive outcomes in PD may differ from those proposed for aerobic exercise and have been discussed in the context of the ‘muscle‐brain axis’ [29]. Resistance exercise can increase the release of myokines and other muscle‐derived factors, which are hypothesised to influence synaptic plasticity and neurogenesis and to interact with systemic metabolic and inflammatory regulation [46–48]. It is through these peripheral adaptations that resistance training may engender systemic conditions that are more conducive to brain plasticity and functional recovery, although direct causal evidence in PD remains limited.

### 5.3. Mind–Body Exercise

Mind–body exercise is defined as a holistic training approach that integrates physical movement, breath regulation, and attentional or mental practices. It is typically characterised by low‐to‐moderate intensity and moderate session duration [56]. A plethora of clinical trials in PD have evaluated the efficacy of Tai Chi, Qigong, dance‐based programmes and meditation‐related practices. The benefits of these programmes have been reported to extend to mood, sleep and cognitive domains. A comprehensive summary of the domain‐specific evidence can be found in Section 4 [36, 57–60]. It is suggested by certain studies that low‐to‐moderate‐intensity mind–body programmes may be particularly relevant for cognitive outcomes. However, comparisons across modalities remain heterogeneous [57].

The application of mind–body interventions in the context of neural plasticity in healthy populations has been a subject of discussion; however, the mechanistic evidence in PD remains limited. A number of comparative analyses have reported larger cognitive effects for mind–body programmes than for aerobic or resistance training, although between‐study differences in dose and outcome measures complicate direct comparisons [61]. In the field of PD, the extant evidence base is predominantly of a clinical nature, given the considerable difficulty of modelling and standardising these interventions in animal models [56].

Mechanistic studies in PD remain limited, and there is a paucity of trials that establish a link between mind–body dose and adherence to objective neural or biomarker readouts. Future research endeavours would benefit from the integration of standardised clinical endpoints with neuroimaging and well‐defined biomarker panels, thereby enabling a more precise correlation between intervention dosage and alterations in brain networks, as well as outcomes in terms of symptoms.

### 5.4. Multimodal Exercise

Multimodal exercise is defined as structured programmes that combine two or more components (most commonly aerobic and resistance training, with balance and flexibility elements when indicated) [29]. In PD, the efficacy of multimodal programmes has been evaluated over periods ranging from weeks to years. These programmes have been associated with improvements in cognition, mood, sleep and quality of life in specific cohorts [62–65]. As outlined in the relevant literature, several trials have reported on biological changes, including neurotrophic factors. However, these measures are not consistently collected, and further research is required to ascertain their clinical relevance [62, 64].

In comparison with single‐mode programmes, the combination of exercise components has been demonstrated to potentially offer a more comprehensive match with the multi‐domain needs of PD, thereby supporting broader functional goals. However, it should be noted that discrepancies in dosage and outcomes across studies hinder the ability to draw definitive comparative conclusions [51]. In clinical practice, exercise is generally delivered in conjunction with standard medical care. However, future trials should aim to provide a more precise definition of co‐interventions (e.g. medication state, rehabilitation add‐ons or device‐based therapies) and assess whether multimodal exercise provides incremental benefits beyond usual care [29, 66–68].

## 6. Impact of Different Exercise Parameters

### 6.1. Impact of Exercise Intensity

Regarding exercise intensity, clinical studies often report larger improvements in fitness and selected clinical outcomes with higher‐intensity prescriptions than with low‐intensity training, although results are heterogeneous across interventions and endpoints [69, 70]. Trials in early PD also suggest that high‐intensity aerobic exercise can be feasible and safely delivered under supervision, supporting its use when tolerated [71]. For example, high‐intensity interval training (HIIT) and moderate‐intensity continuous training (MICT) cycling programmes have both been evaluated in PD, with some studies reporting improvements in cognitive performance and suggesting that response magnitude may relate to the achieved training intensity [52, 72]. However, direct comparisons indicate that HIIT does not uniformly outperform MICT for motor or non‐motor outcomes, despite potential advantages for cardiorespiratory fitness in some cohorts [73–75]. Higher‐intensity training may also drive larger adaptations in cardiorespiratory fitness, cerebrovascular and metabolic regulation and systemic inflammatory tone, which could plausibly contribute to non‐motor outcomes and overall function [71, 72].

Given the variability in motor impairment, comorbidity and fall risk, intensity should be individualised according to disease stage, baseline fitness and safety considerations, with progression guided by tolerability and objective monitoring (e.g. HR or perceived exertion).

### 6.2. Exercise Cycle

Exercise interventions in PD span a wide range of durations, from single‐session paradigms to multi‐month and multi‐year programmes. Evidence from Tai Chi cohorts suggests that sustained participation across 12‐week to multi‐year periods can be associated with improvements in neurocognitive and affective outcomes, although reported effects vary by study design and endpoints [37, 76, 77]. The importance of maintenance is further emphasised by the findings of a more extensive follow‐up study. A 6‐year longitudinal study reported that improvements in depressive symptoms and quality of life emerged after a short initial intervention and were sustained over subsequent follow‐up, with continued participation linked to better long‐term maintenance of benefits [78]. Furthermore, the implementation of repeated short‐term high‐intensity training blocks over an extended period has been utilised as a maintenance strategy. This approach has yielded consistent or favourable clinical outcomes in certain cohorts over an extended timeframe [79]. Observational evidence lends further support to the overarching notion that the maintenance of regular physical activity and exercise routines is associated with a more favourable clinical progression in PD [80].

Evidence from a number of studies suggests that a single exercise session can result in quantifiable, short‐term alterations in neurophysiological markers and learning‐related outcomes. For instance, a 20‐minute session of moderate‐intensity aerobic exercise has been associated with alterations in cortical motor excitability, as measured by transcranial magnetic stimulation [81]. Acute sessions have also been reported to increase serum BDNF in PD [82], and moderate‐intensity cycling has been associated with improvements in motor memory and indices of neural plasticity [83, 84]. These acute responses may offer valuable insights into plasticity; however, further elucidation is necessary to elucidate their association with sustained non‐motor symptom change.

The current body of evidence suggests that sustained participation and planned progression may be pivotal in maintaining exercise‐related benefits in PD. However, significant variations in dosage, adherence and outcome assessment impede the capacity for meaningful comparisons across programmes of varying durations. It is recommended that future studies implement a standardised approach to the reporting of programme length, maintenance approaches and post‐intervention follow‐up. This would serve to clarify the durability of the observed effects [3].

## 7. Summary and Prospect

Exercise is increasingly recognised as an important adjunctive strategy in PD, with growing evidence of benefits across several non‐motor symptom (NMS) domains, including affective disturbances, cognitive dysfunction, sleep disorders, pain and autonomic abnormalities. However, the magnitude of these effects can vary according to the exercise modality employed. Furthermore, autonomic outcomes are not assessed with the same degree of consistency as other domains. Differences in exercise dose, participant characteristics, and study design limit the comparability of findings across studies and make the definition of symptom‐specific prescriptions difficult. From a mechanistic perspective, the current findings are consistent with an integrative peripheral–central model in which exercise‐related cardiometabolic and immunometabolic adaptations, in conjunction with myokine release, interact with central neuroimmune regulation, network plasticity and cerebrovascular/metabolic support within circuits linked to NMS. However, a significant proportion of the mechanistic evidence is of an associative nature, and more explicit links between exercise‐driven biological changes and improvements in specific NMS are still required.

Future research should prioritise standardised NMS phenotyping (e.g. autonomic measures), precise reporting of exercise dose and adherence and longer follow‐up to evaluate durability. The combination of clinical trials with multimodal mechanistic measures, including neuroimaging, neurophysiology and multidimensional biomarker profiling, will facilitate the mapping of symptom‐relevant pathways and elucidate the neurobiological basis of exercise‐related improvements in PD.

## Author Contributions

**Shuang Hu**: writing – original draft, investigation, formal analysis. **Jing Lei**: writing – review and editing, writing – original draft, supervision, methodology, investigation, funding acquisition, formal analysis, conceptualisation. **Hao-Jun You**: writing – review and editing, writing – original draft, supervision, methodology, investigation, funding acquisition, formal analysis, conceptualisation.

## Funding

This research was supported by the National Natural Science Foundation of China (Grants 82472612, 82074564 and 81860410).

## Conflicts of Interest

The authors declare no conflicts of interest.

## Data Availability

The data that support the findings of this study are available from the corresponding author upon reasonable request.
